# Comment on Kaufman et al. Popular Dietary Trends’ Impact on Athletic Performance: A Critical Analysis Review. *Nutrients* 2023, *15*, 3511

**DOI:** 10.3390/nu15224709

**Published:** 2023-11-07

**Authors:** Robert Wakolbinger-Habel, Christian Muschitz

**Affiliations:** 1 Department of Physical and Rehabilitation Medicine (PRM), Vienna Healthcare Group—Clinic Donaustadt, Academic Teaching Hospital of the Medical University of Vienna, A-1220 Vienna, Austria; 2 HealthPi Medical Center, 1010 Vienna, Austria

The authors [1] cite our paper “Self-reported Resistance Training Is Associated With Better HR-pQCT–derived Bone Microarchitecture in Vegan People” published in The *Journal of Clinical Endocrinology & Metabolism* in 2022 [2].

They state that in our study, “vegan athletes had bone quality compared to omnivores and had decreased benefit in bone quality from resistance training than omnivores as well”.

In fact, we obtained different findings. In our study, vegans performing resistance training at least once a week (self-reported) not only had significantly better bone structure than vegans not performing resistance training, but also there were hardly any differences in bone structure parameters compared to omnivores performing resistance training.

In addition, according to our data, the benefit from resistance training might be even stronger (not decreased) when adhering to a vegan diet, as the numeric differences in bone structure parameters were larger between the two vegan subgroups than between the two omnivore subgroups.

In summary, we observed similar bone structure in both the vegan resistance training active group and the omnivore resistance training active group.
